# Difference in phenotypic severity of presumed null alleles of *capg-1*

**DOI:** 10.17912/micropub.biology.000245

**Published:** 2020-05-10

**Authors:** Sarah VanDiepenbos, Györgyi Csankovszki

**Affiliations:** 1 Department of Molecular, Cellular, and Developmental Biology, University of Michigan, Ann Arbor, MI 48109 USA

**Figure 1:**
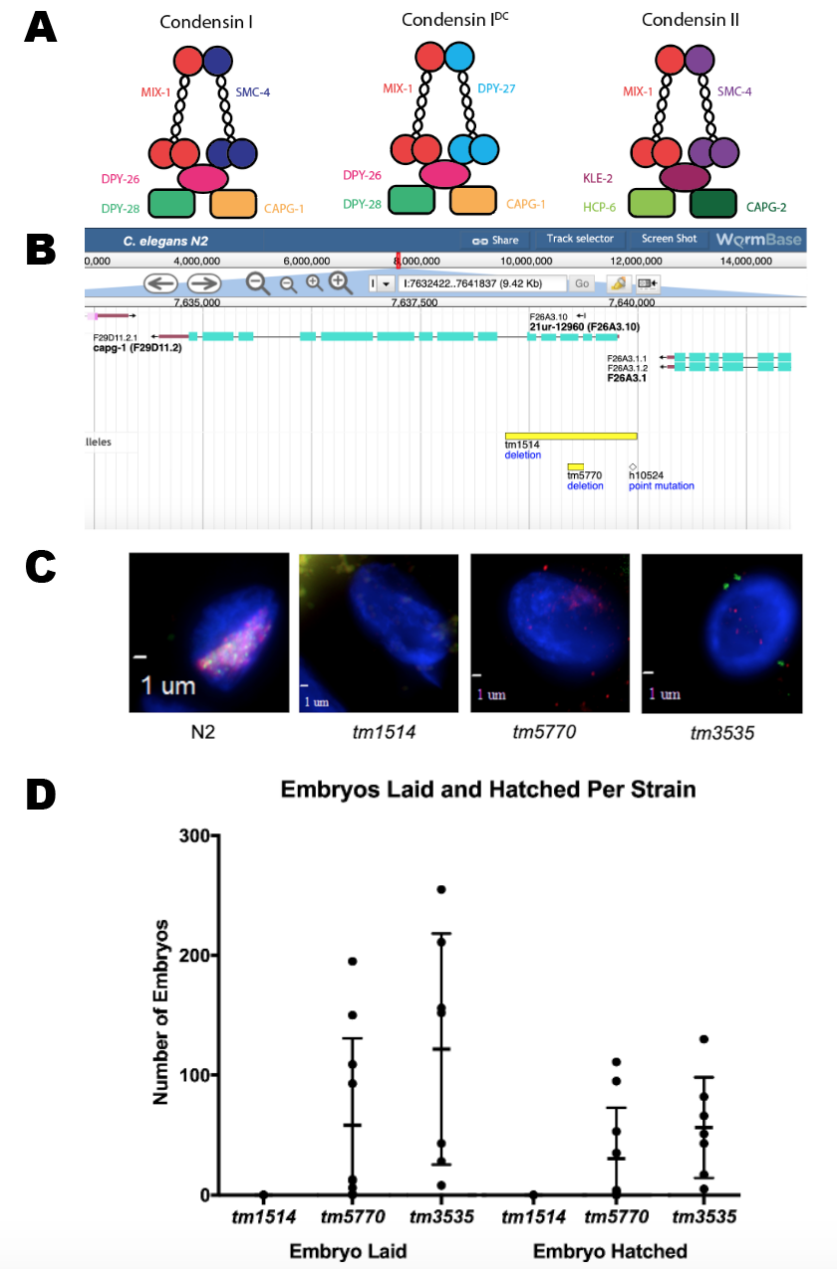
**A**. Subunit composition of condensin I, condensin I^DC^, and condensin II complexes. DPY-28 (green), DPY-26 (pink), and CAPG-1 (light orange) are found in both condensin I and condensin I^DC^. **B**: *capg-1* gene information obtained from WormBase, showing the *tm1514* deletion, *tm5770* deletion, and the 21ur-12960 piRNA. **C:** Immunofluorescence images of adult hermaphrodite intestinal nuclei in wild type N2, *capg-1(tm1514)*, *capg-1(tm5770)*, and *dpy-28(tm3535)* stained for DPY-27 (with Cy3, red) and CAPG-1 (with FITC, green), merged with DAPI. X-localized DCC is only seen in N2. **D:** Number of embryos laid and hatched in each strain. Each dot represents the number of embryos laid by an individual worm. The *capg-1(tm1514)* mutants (n=10) did not lay any eggs, while both *capg-1(tm5770)* mutants (n=10) and *dpy-28(tm3535)* mutants (n=7) laid a significant number of eggs, many of which hatched.

## Description

Dosage compensation is the mechanism by which organisms correct the sex chromosome imbalance between sexes (e.g. females having two X chromosomes compared to one X in males). In *C. elegans*, dosage compensation is achieved by the downregulation by half of both X chromosomes in hermaphrodites (Albritton & Ercan, 2018). This downregulation is accomplished by the Dosage Compensation Complex (DCC), which is comprised of a condensin I^DC^ subcomplex interacting with other accessory proteins. Condensin I^DC^ has a similar structure to canonical condensins (condensin I and condensin II), which function to compact chromosomes during mitosis and meiosis (Csankovszki *et al.*, 2009). The DPY-27 subunit is unique to condensin I^DC^, MIX-1 is present in all three condensins, while the proteins CAPG-1, DPY-26, and DPY-28 are found both in condensin I and condensin I^DC^ (Figure 1A).

DCC mutants show maternal effect lethality, since all subunits of condensin I^DC ^and several of the accessory proteins are maternally contributed to oocytes (Plenefisch *et al.*, 1989; Csankovszki *et al.*, 2009). Homozygous DCC mutants derived from heterozygous mothers survive to adulthood due to the maternally provided RNA and/or protein. These mutants are referred to as maternal positive, zygotic negative (M+Z-). M+Z- hermaphrodites are unable to produce a functional gene product; therefore, their progeny have no maternal or zygotic contribution of these proteins (M-Z-). As a consequence, very few M-Z- hermaphrodites survive past the L1 stage; however, males do not require the DCC to survive. It is possible, then, to recover M-Z- male progeny from self-fertilizing hermaphrodites in these conditions.

A previous study (Csankovszki *et al.* 2009) showed that M+Z- *capg-1* null mutants (*tm1514*) are sterile and have severe developmental defects. This phenotype is different from and more severe than what was previously seen for genes encoding other condensin I^DC^ members (Plenefisch *et al.*, 1989). It raised the possibility that the sterility and more severe developmental phenotypes of *capg-1*(*tm1514*) is the result of another role of CAPG-1 outside of condensin I and I^DC^ function. We acquired another *capg-1* allele (*tm5770*) from the Japanese National Bioresource Project. This allele deletes a smaller portion of the coding sequence than *capg-1*(*tm1514*) but is also predicted to be null due to the resulting frameshift mutation. Also of interest, the *capg-1*(*tm5770*) allele removes only the terminal nucleotide from a short piRNA gene deleted entirely in *capg-1*(*tm1514*) (Figure 1B). If the absence of the CAPG-1 protein function was responsible for the sterility phenotype observed in *capg-1*(*tm1514*), the M+Z- hermaphrodites from the *capg-1*(*tm5770*) strain should also be sterile.

We first confirmed via fluorescence microscopy that the DCC is not recruited to the X chromosome in the *capg-1*(*tm1514*) adult hermaphrodites, consistent with previously published results (Csankovszki *et al.*, 2009). For additional control, we used a mutation in another condensin I^DC^ member, *dpy-28(tm3535)*, which has similar defects(Hernandez *et al.*, 2018)*.* Fluorescent antibodies specific to CAPG-1 and another condensin I^DC^ subunit, DPY-27, were used to visualize localization of the DCC to the X chromosome compared to wild type (N2) (Figure 1C). N2 hermaphrodites have overlapping signals of CAPG-1 and DPY-27 on both X chromosomes. The X localization of these two condensin I^DC^ proteins is also absent in *capg-1*(*tm5770*). This indicates that the *capg-1*(*tm5770*) mutation also disrupts DCC localization to the X to a similar degree as *capg-1(tm1514)* or *dpy-28(tm3535)*.

The *capg-1*(*tm5770*) M+Z- hermaphrodites, unlike the *capg-1(tm1514)* mutants, were observed laying embryos, some of which hatched then arrested in L1, showing more phenotypic similarity to the *dpy-28(tm3535)* mutants than the *capg-1(tm1514)* mutants. To quantify this observation, we conducted embryo and lethality counts in *capg-1(tm1514)*, *capg-1(tm5770)*, and *dpy-28(tm3535)* mutants to assess both the number of embryos laid and the number of embryos hatched (Figure 1D). Our results show that while *capg-1(tm1514)* M+Z- mutants did not lay any eggs, the *capg-1(tm5770)* M+Z- and the *dpy-28(tm3535)* M+Z- mutants produced significant numbers of embryos. Many of the embryos laid by the *capg-1(tm5770)* and *dpy-28(tm3535)* mutants hatched then arrested in the L1 stage. Interestingly, there is a high amount of variability in numbers of embryos laid between individual worms in the *dpy-28(tm3535)* and *capg-1(tm5770)* strains. The *dpy-28(tm3535)* and *capg-1(tm5770)* mutants produced a small percentage of M-Z- progeny that survived until adulthood. Phenotypically, these were either males or very Dpy hermaphrodites that had severe developmental defects and were sterile. The *capg-1(tm5770)* mutants (n=10) laid an average of 58 embryos per worm, ranging between 0 and 195. Of the 580 total embryos laid, 297 hatched, of which 291 arrested in the L1 stage, with 1 male and 5 hermaphrodites surviving to adulthood. The *dpy-28(tm3535)* mutants (n=7) laid an average of 122 embryos per worm, ranging between 8 and 255. Of the 853 total embryos laid, 354 hatched, of which 340 arrested in the L1 stage, with 9 males and 5 hermaphrodites surviving to adulthood. The appearance of males in the M-Z- progeny is consistent with a weak Him phenotypereported previously for condensin I mutants (Plenefisch *et al.*, 1989; Hernandez *et al.*, 2018). Overall, these results indicate that the *capg-1(tm5770)* mutation results in phenotypes resembling the phenotypes caused by *dpy-28(tm3535)* and other mutations in condensin I^DC^ subunits (Plenefisch et. al, 1989). These condensin I^DC^ mutant phenotypes are different from the phenotypes resulting from the *capg-1(tm1514)* mutation.

These data suggest that the more severe phenotype in the *capg-1(tm1514)* mutants is not due to disruption of CAPG-1 function. There are several potential alternative explanations. The phenotype may be due to the deletion of the piRNA gene near the 5’ end of the *capg-1* gene (Figure 1B). It is also possible that the severe phenotype observed in *capg-1(tm1514)* is due to a disruption of the *trans*-splice site between genes. The *capg-1* gene is last in its operon, and the *capg-1(tm1514)* deletion includes a *trans*-spliced acceptor site (Worm Base). This would result in defective *trans*-splicing between *capg-1* and the upstream gene, F26A3.1. Our data does suggest, however, that the severity of the *capg-1(tm1514)* phenotype is not due to an alternative role of CAPG-1 outside of condensin I and condensin I^DC ^function.

## Methods

Strains: All *C. elegans* strains were maintained using standard methods and fed *E. coli* (OP50) on NG agar plates and maintained at 20^o^C. The strains used included N2 Bristol strain (wild-type) as a negative control, EKM4 *capg-1(tm1514)* I*/hT2 [qIs48]* (I;III), EKM86 *capg-1(tm5770)* I*/hT2[qIs48]* (I;III), and EKM40 *dpy-28(tm3535)* III*/hT2[qIs48]* (I;III). M+Z- hermaphrodites were identified by selecting GFP-negative progeny of GFP-positive hermaphrodites.

Immunofluorescence Imaging: Young adult worms were dissected with needles in 10μL of 1X sperm salts (50mM Pipes pH7, 25 mM KCl, 1 mM MgSO4, 45 mM NaCl, with 1 mM levamisole as a sedative), fixed in 2% paraformaldehyde in 1X sperm salts for five minutes in a humid chamber moistened with PBST (PBS with .1% Triton X-100), and frozen on dry ice with a coverslip for at least 15 minutes. After freezing, the coverslip was carefully separated with a razor blade and the slides were washed three times for 10 minutes each in PBST. This was followed by overnight incubation in a humid chamber with 40μL of a solution of primary antibodies rabbit anti-DPY-27 and rat anti-CAPG-1 (Csankovszki *et al.*, 2009) diluted 1:250 in PBST. One primary antibody targeted DPY-27, which is part of Condensin I^DC^ in the Dosage Compensation Complex, and was raised in rabbit (Csankovszki *et al.*, 2009). The other primary antibody targeted CAPG-1, which is part of both Condensin I and Condensin I^DC^, and was raised in rat. Incubation with primary antibody was overnight in a humid chamber at room temperature. The next day, slides were washed three times for 10 minutes each in PBST, incubated for 1 hour at 37^o^C with 30μL of a solution of secondary antibody (Jackson Immunochemicals Cy3 conjugated anti-rabbit for DPY-27 and FITC conjugated anti-rat for CAPG-1 at 1:100), and washed again three times for 10 minutes each in PBST with the final wash containing 1uL of DAPI (1mg/mL). Slides were then mounted with Vectashield (Vector Laboratories).
